# The gut microbial differences between pre-released and wild red deer: Firmicutes abundance may affect wild adaptation after release

**DOI:** 10.3389/fmicb.2024.1401373

**Published:** 2024-07-15

**Authors:** Jinhao Guo, Zheng Li, Yongchao Jin, Yue Sun, Binying Wang, Xinxin Liu, Ziao Yuan, Weiqi Zhang, Changzhi Zhang, Minghai Zhang

**Affiliations:** ^1^College of Wildlife and Protected Area, Northeast Forestry University, Harbin, China; ^2^College of Biological Sciences and Technology, Beijing Forestry University, Beijing, China; ^3^Forestry and Grassland College, Jilin Agricultural University, Changchun, China; ^4^World Wild Fund for Nature, Beijing, China; ^5^School of Biological Sciences, Guizhou Education University, Guiyang, China; ^6^College of Life Science and Technology, Harbin Normal University, Harbin, China; ^7^Forestry College, Jiangxi Environmental Engineering Vocational College, Ganzhou, China

**Keywords:** red deer, gut microbes, wild release, population recovery, feeding adaptation

## Abstract

Insufficient density of red deer has affected the stability of forest ecosystems and the recovery of large carnivores (represented by Amur tiger). Conservation translocations from captivity to the wild has become an important way to restore the red deer populations. However, the difference in gut microbes between pre-release and wild red deer may affect the feeding adaptability of red deer after release. In this study, we clarified the differences in gut microbes between pre-released and wild red deer and screened the key gut microbes of the red deer involved in feeding by using metagenomic sequencing and feeding analysis. The results showed that the microbial difference between pre-released and wild red deer was mainly related to Firmicutes represented by Eubacteriales and Clostridia, and Firmicutes abundance in pre-released red deer (68.23%) was significantly lower than that of wild red deer (74.91%, *p* < 0.05). The expression of microbial metabolic pathways in pre-released red deer were significantly lower than those in wild red deer (*p* < 0.05), including carbohydrate metabolism, amino acid metabolism, glycan biosynthesis and metabolism, etc. The combinations of Firmicutes were significantly positively correlated with the intake of plant fiber and carbohydrate (*p* < 0.05), and were key microbes to help red deer deal with wild plant resources. Additionally, the combinations of Firmicutes represented by Eubacteriales and Clostridia lacking in pre-released red deer contributed the most to expression of microbial metabolic pathways (*importance* > 1), showing a significant positive correlation (*p* < 0.05). This study indicates that high abundance of Firmicutes is an important guarantee for red deer to adapt to the wild feeding environment, which provides critical implications for the recovery of red deer populations and the protection of endangered ungulates.

## 1 Introduction

Feeding constitutes an essential link between ungulates and the habitat, which is important evidence to measure the wild adaptability of ungulates (Zhang and Li, 2008; Raubenheimer et al., 2009). In the reintroductions and translocations of endangered animals for population recovery, the focus of research is to improve the feeding adaptability after release (IUCN/SSC, 2013). The ungulates mainly feed on high-fiber plants in the wild, and the decomposition of high-fiber components mainly depends on fiber degrading bacteria in the gut (Flint et al., 2015; Kameshwar and Qin, 2016). Therefore, gut microbes are related to the feeding adaptation of ungulates. While feeding affects the gut microbial community (Muegge et al., 2011; Jiang et al., 2020; Huang et al., 2021), gut microorganisms provide nutrients needed by ungulates by decomposing various substances in plants (Buddington and Sangild, 2011). Given the important role of gut microbes in feeding, it is important to identify the differences in gut microbes between pre-released and wild individuals and understand the core gut microbes associated with handling wild plant resources for improving the environmental adaptability after release.

Red deer (*Cervus elaphus*) is one of the typical ungulates in the natural forest area of northern China, which plays an important role in promoting the stability of forest ecosystems and the recovery of large carnivores (represented by Amur tiger *Panthera tigris altaica*), and has high research and protection value (Sheng, 1992; Niu et al., 2021). At the end of the 20th century, red deer declined sharply due to weak protection awareness and the economic development of forest areas (Xu et al., 2000). Although the establishment of nature reserves has effectively promoted the recovery of wild red deer populations, the recovery rate is slow (Soh et al., 2014). A series of studies are urgently needed to protect and restore red deer population. Wild release is gradually becoming a conservation method for wild population recovery, and captive populations have become an important source of wildlife reintroductions and translocations (IUCN/SSC, 2013; Guo et al., 2022).

The difference in gut microbes between captive and wild populations may reduce the adaptability of captive populations to the wild environment after release (Cheng et al., 2020). At present, the microbial difference between pre-released and wild red deer is unknown, and the important microbial community related to the processing of complex plant resources in the wild needs to be further studied. Understanding the differences in gut microbes between pre-released and wild red deer and identifying important metabolic microbes can provide an important theoretical reference for improving the success rate of wild release, and promote the theoretical study of gut microbes-feeding adaptation mechanism.

The Gaogesitai Nature Reserve, located in the southern of the Greater Khingan Mountains of China, is an important source base for the wild release of red deer, with captive populations and a certain number of wild populations, providing an opportunity to compare the differences in gut microbes between pre-released and wild red deer.

In this study, we compared the actual differences in gut microbes between pre-released and wild red deer through metagenomic sequencing technology, and further identified important microbial groups related to adaptation to feeding changes, with a view to providing theoretical reference for future wild release work of red deer.

## 2 Materials and methods

### 2.1 Study area and fecal sample collection

**Figure 1:**
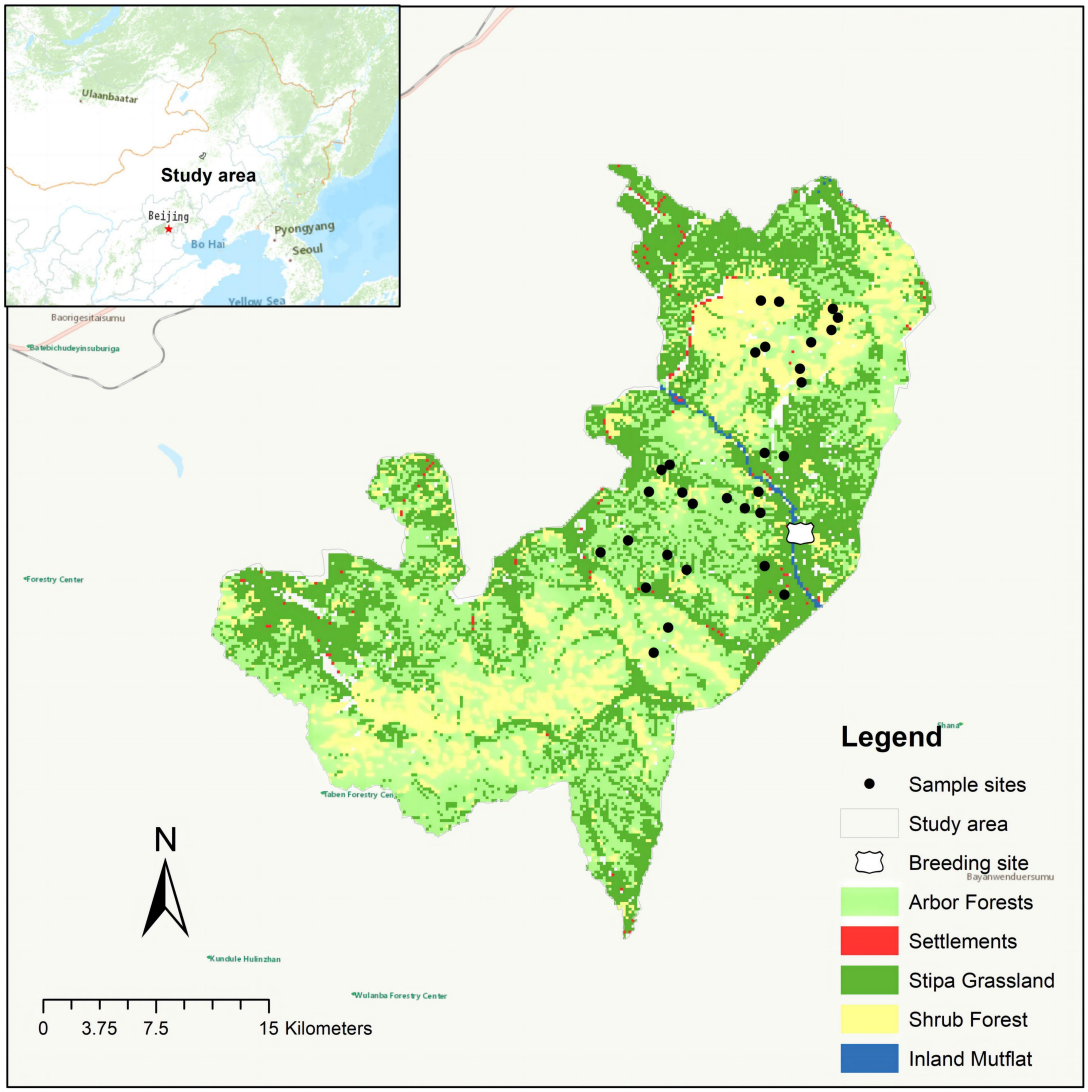
A map showing the locations of the study area and sampling.

The Gaogesitai National Nature Reserve is in the southern of the Greater Khingan Mountains, with a total area of 1062.84 km^2^ (Figure 1). The vegetation in the area is mainly mountain forest and shrubs, with a mosaic distribution of stipa grass. The mountain forest is mainly deciduous broad-leaved forest, including *Ulmus pumila*, *Quercus mongolica*, and *Betula platyphylla*. The main mountain shrubs are *Salix rosmarinifolia*, *Ostryopsis davidiana*, *Prunus sibirica*, and *Rhododendron dauricum*. The grassland vegetation is mainly Gramineae, Cyperaceae, and Asteraceae. The special forest-grassland transition zone landscape in the protected area provides unique habitat and food resources for the survival and reproduction of red deer, which is the main base for the recovery of the red deer population in China.

Fresh fecal samples of pre-released and wild red deer were collected in December 2022 (the information of pre-released red deer was shown in Supplementary Table S1). The collection of fecal samples in the wild was completed by tracking footprint chains. After the samples were confirmed as fresh feces, the uncontaminated part of the fecal sample was collected using disposable PE gloves. New PE gloves were replaced each time to prevent cross-contamination between samples. Since wild red deer mainly ate arbors, shrubs, and herbs (Sun, 2019), all collections have been carried out in these three habitats. A total of 32 fresh feces samples of wild red deer were collected, 22 different wild red deer individuals were identified with 8 pairs of highly polymorphic microsatellite primers (ETH225, T501, T156, BM848, T530, DM45, N, T507; Tian, 2021). And the fecal samples derived from different wild individuals were randomly divided into two parts for microbial differential comparison between pre-released and wild red deer (*n* = 12), and association analysis of gut microbes and feeding (*n* = 10), each samples’ part covered the entire habitats the wild red deer distributed. All fecal samples were quickly frozen by using liquid nitrogen, and dry ice was used to transport fecal samples back to the laboratory and preserved at −80°C.

### 2.2 Survey of available plants of red deer

The investigation and collection of available plant resources of red deer were carried out. When red deer activity traces (feces, urine, lying traces, and gnawing traces) were found, 10 m × 10 m plant quadrats were set with red deer activity traces as the center point, and small quadrats of 2 m × 2 m were set at the four corners and center of the quadrats. The species and quantity of arbors and shrubs in the large quadrats and the species and coverage of herbs in the small quadrats were recorded, and non-repetitive vegetation was collected for feeding analysis and determination of nutrient composition.

### 2.3 Feeding and nutritional analysis

Fecal microscopy technology was used to compare plant fragments in wild red deer feces with known plant fragments, and the occurrence frequency of plant fragments was calculated (Zhong, 2021). The frequency conversion method was used to convert the frequency of each plant fragment into the average density of fragments of available plants by red deer. The average density was converted to the relative density of recognizable plant fragments (Sparks and Malechek, 1968). The micro-histological analysis did not affect the ranking results of feeding components (Henley et al., 2001; Shrestha and Wegge, 2006). Therefore, the feeding results obtained by micro-histological techniques were comparable for the same animal.

The contents of crude protein (CP), crude fat (CF), neutral detergent fiber (NDF), acid detergent fiber (ADF), acid detergent lignin (ADL), ash and tannin in available plants of red deer were determined (Zhang, 2007). The contents of non-structural carbohydrate (NSC), carbohydrate (CHO), and digestible fiber (DF) were calculated based on the results of the above determination (Yuan et al., 2022):

NSC% = [100 − (NDF% + CP% + Ash% + CF%)]

CHO% = NDF% + NSC%

DF% = NDF% − ADL%

The intake of different nutrients was calculated (Yuan et al., 2022):


$$DM=\overset{n}{\underset{i}{\sum }}RD_{i}\times N_{i}$$

RD_i_ was the proportion of plant species i in the feeding structure, and N_i_ was the concentration of nutrient N in plant species i (% dry matter weight).

### 2.4 Metagenomic sequencing

Total DNA of the microbiome in fecal samples was extracted using the Mag-Bind Soil DNA Kit (OMEGA, USA). The concentration and purity of the extracted DNA were tested by Qubit 4 fluorometer (Invitrogen, USA), and the quality of DNA was measured by 1% agarose gel electrophoresis. All DNA concentrations were higher than the minimum concentration requirements for sequencing (>2.5 ng/μl). The total amount of DNA at least met the requirements for single sequencing (>0.2 μg), and the DNA quality fully met the requirements for subsequent analysis. The double-ended sequencing was performed with Illumina NovaSeqTM X Plus sequencing platform (2 × 150 bp).

The original sequence was screened and filtered using fastp software (v0.23.2) to remove some low-quality sequences. After quality screening, sequences (length < 50 bp) and those containing fuzzy bases were removed. The fastp software (v0.23.2) was used to conduct quality statistics of the sequencing data after quality control. The base mass of the sequences used in this study was greater than 20, which met the quality requirements of subsequent data analysis. The minimap2 software (v2.24-r1122) was used to compare the effective sequences after quality control with host sequences, discarded the sequences with a genome mapping score greater than or equal to 10, and used the final sequences with host contamination removed for subsequent analysis.

### 2.5 Data processing and analysis

The Kraken2 software (v2.0.8-beta) was used to annotate the final reads. The MMseqs2 software (v13.45111) was used to compare the filtered protein sequences with the functional databases of KEGG and CAZy, then the functions of gut microbes of red deer were annotated.

Based on the principal component analysis of the bray_curtis distance algorithm, the differences in gut microbes between pre-released and wild red deer were described, and the differences between groups were further evaluated with ANOSIM difference analysis. The STAMP difference analysis and the LEfSe test were performed to compare relative abundance of gut microbes between different groups. The MetagenomeSeq analysis was used to identify the microbial functional pathways with abundance differences. The microbial biomarkers between pre-released and wild red deer were evaluated by using random forest analysis. The feeding and microbial composition of red deer were analyzed based on the bar chart, and the corresponding relationship between feeding and gut microbial structure and function of red deer was analyzed using correlation heat map. In combination with ape and vegan packages of R software (v4.3.2), the functional contributions of microbial community were analyzed to determine important functional microbes related to metabolism (Gower, 1975). And Linear regression analysis was used to identify the correlation between functional expression and the abundance of important functional microbes.

## 3 Data availability

The raw sequences of metagenomic sequencing for this study can be found in the Sequence Read Archive from NCBI (Bio project: PRJNA1087561).

## 4 Results

### 4.1 Differences in gut microbes between pre-release and wild red deer

**Figure 2:**
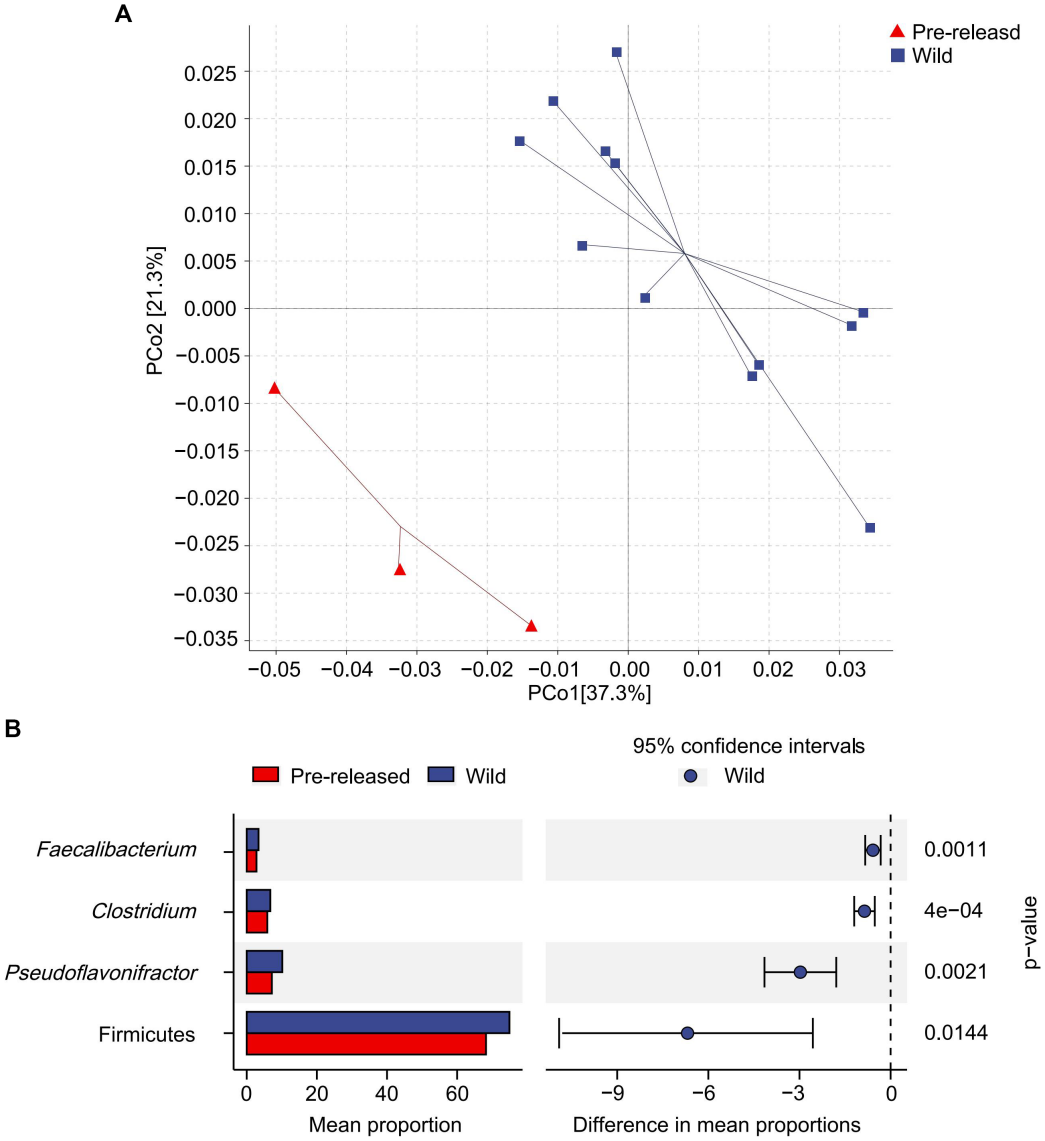
Difference in microbial structure and composition between pre-released and wild red deer. **(A)** The PCoA plot based on Bray-Curtis distance metrics in relation to whole dataset to describe the microbial structure between pre-released and wild red deer. (PCoA1 = 37.3%, PCoA2 = 21.3%); **(B)** the STAMP analysis of gut microbes with abundance differences between pre-released and wild red deer (*p* < 0.05).

**Figure 3:**
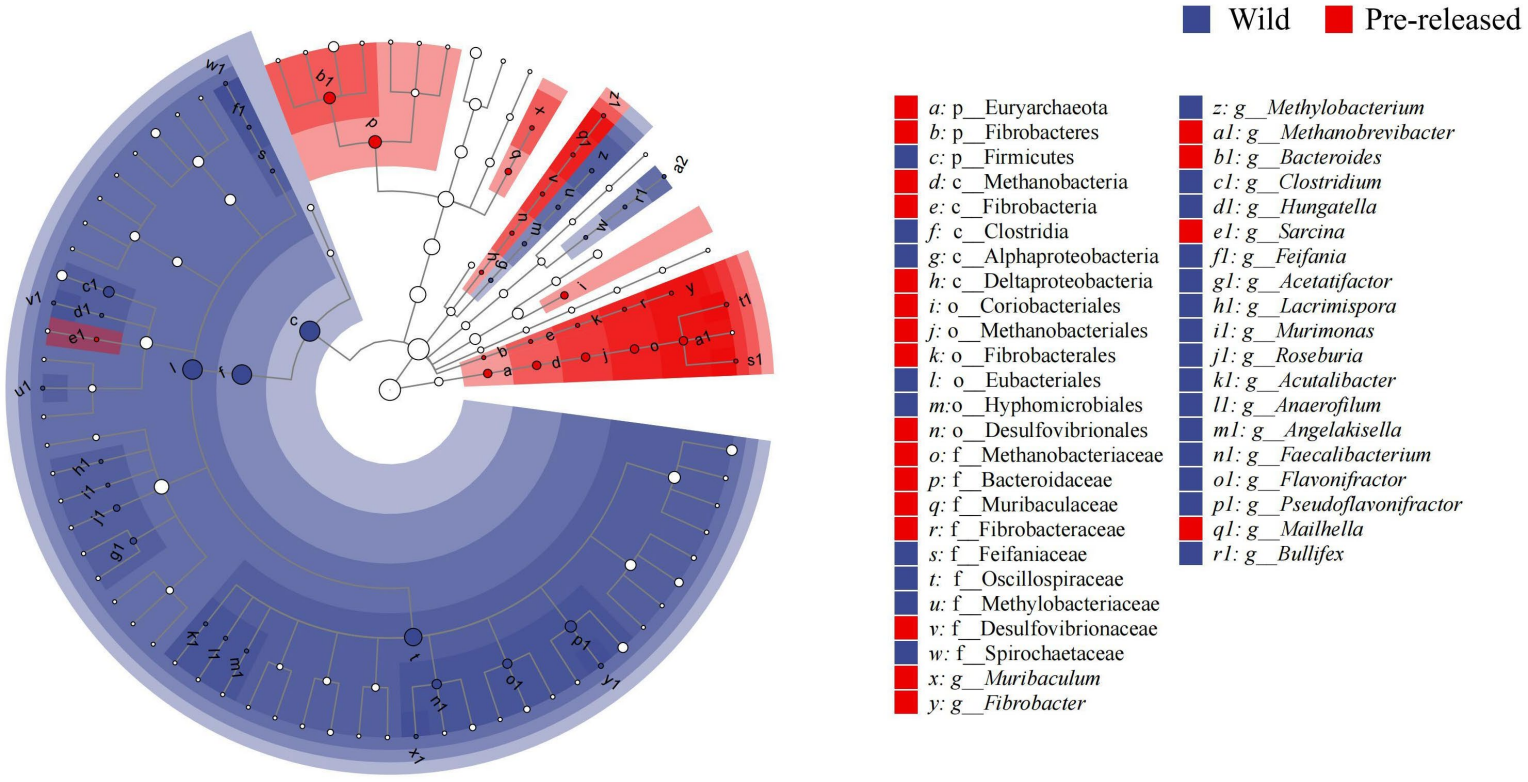
The LEfSe difference test in the microbial communities between pre-released and wild red deer (*p* < 0.05). Colors represent different groups.

A total of 1,028,966,468 (68,597,765 reads/sample) valid sequence were obtained. And 11,361,528 splicing sequences (757,435 reads/sample) were obtained with the average sequence length 300 ~ 210,653.13. The results of PCoA and ANOSIM analysis showed significant differences in microbial structure between pre-release and wild red deer (Figure 2A). At the phylum and genus levels, the differences between groups were significantly more significant than within groups (ANOSIM: phylum: *p* = 0.003, *R* = 0.91; genus: *p* = 0.003, *R* = 0.95). The combinations of Firmicutes were responsible for the difference in gut microbes between pre-released and wild red deer (*p* < 0.05, Figures 2B, 3). Compared to wild red deer (74.91%), Firmicutes were significantly lower in pre-released red deer (68.23%, *p* < 0.05). The low abundance of Firmicutes in pre-released red deer was mainly related to the low abundance of Eubacteriales and Clostridia (*p* < 0.05, Figure 3).

**Figure 4:**
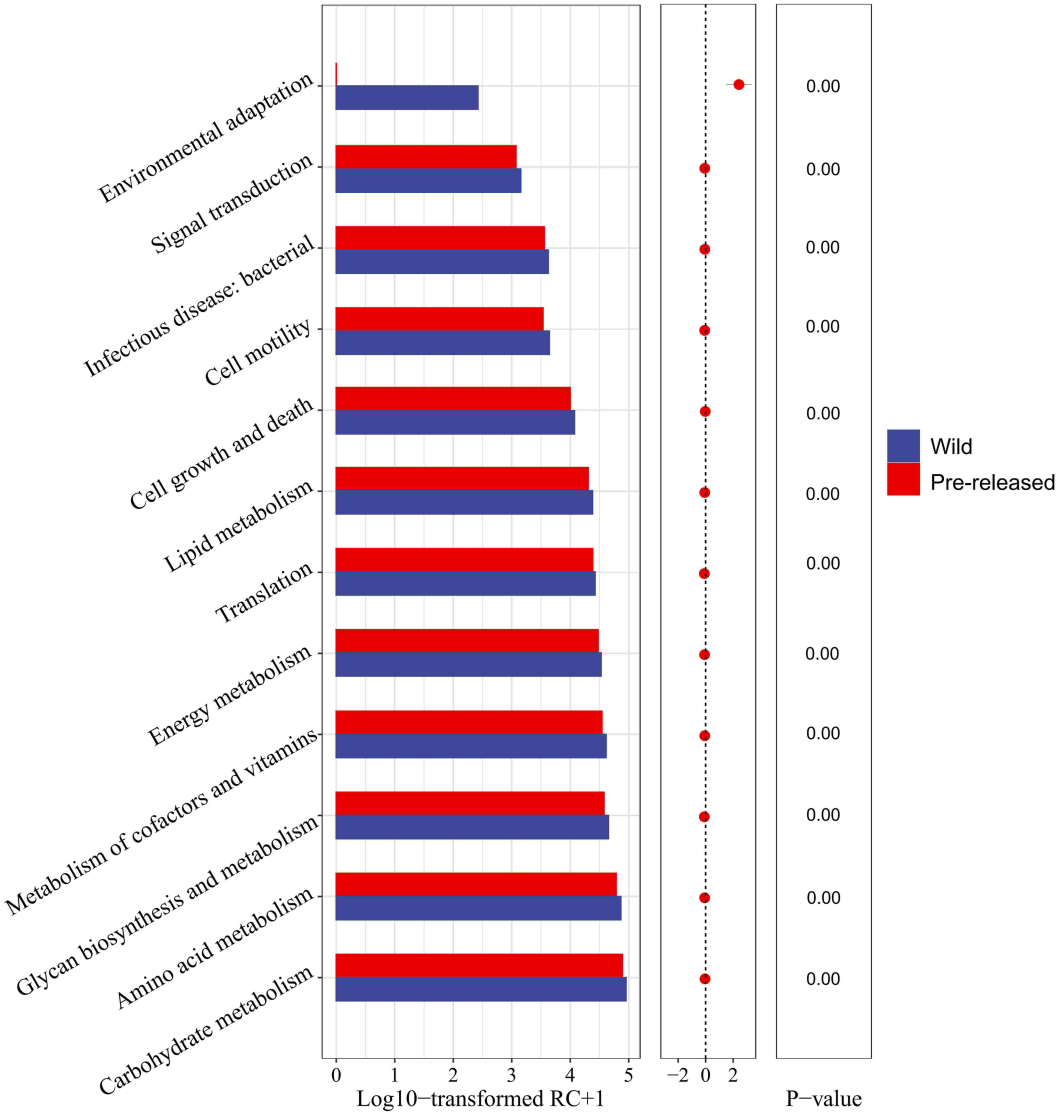
Difference in the expression of microbial functions between pre-released and wild red deer (*p* < 0.05). Colors represent different groups.

A further comparison of the microbial functions in pre-release and wild red deer found that among the sub-pathways involved in metabolism, carbohydrate metabolism, amino acid metabolism, glycan biosynthesis and metabolism, metabolism of cofactors and vitamins, energy metabolism, and lipid metabolism in pre-release red deer were significantly lower than that of wild red deer (*p* < 0.05). Among the sub-pathways related to environmental information processing, environmental adaptation in pre-release red deer was significantly lower than that of wild red deer (*p* < 0.05, Figure 4).

### 4.2 The microbial community related to feeding of red deer

**Figure 5:**
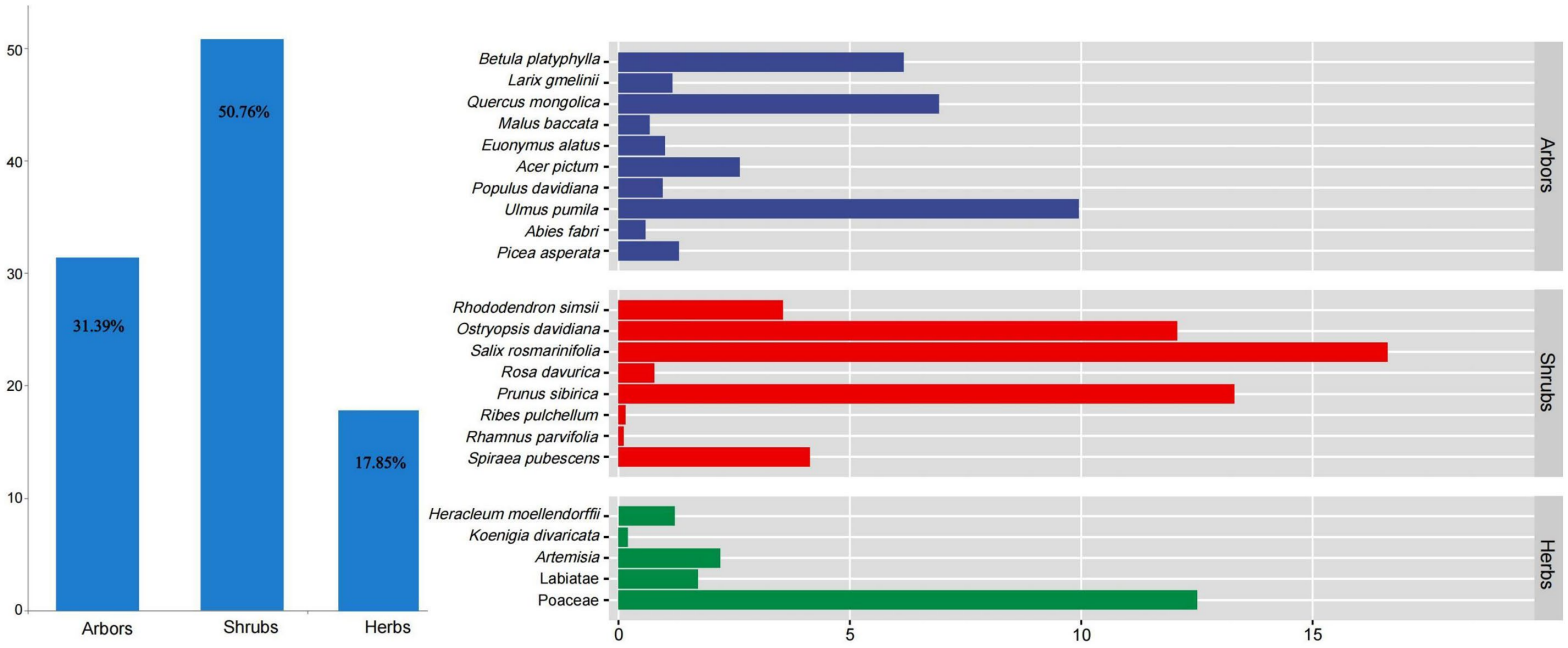
The feeding structure of red deer in the wild. Colors represent different plant types fed by red deer.

Shrubs were the main food sources for red deer in the wild (RD% = 50.76%), followed by arbors (RD% = 31.39%) and herbs (RD% = 17.85%, Figure 5). Among them, *Salix rosmarinifolia* (RD% = 16.62%), *Prunus sibirica* (RD% = 13.31%), and *Ostryopsis davidiana* (RD% = 12.08%) were the main feeding shrubs. *Ulmus pumila* (RD% = 9.95%), *Quercus mongolica* (RD% = 6.93%), and *Betula platyphylla* (RD% = 6.17%) were the main feeding arbors. Poaceae (RD% = 12.5%) was the main feeding herbs.

The average intake of CP of wild red deer was 5.45%, the average intake of CF was 5.63%, the average intake of plant fiber was 52.64%, the average intake of tannin was 1.07%, the average intake of NSC was 31.99%, and the average intake of CHO was 84.63%.

**Figure 6:**
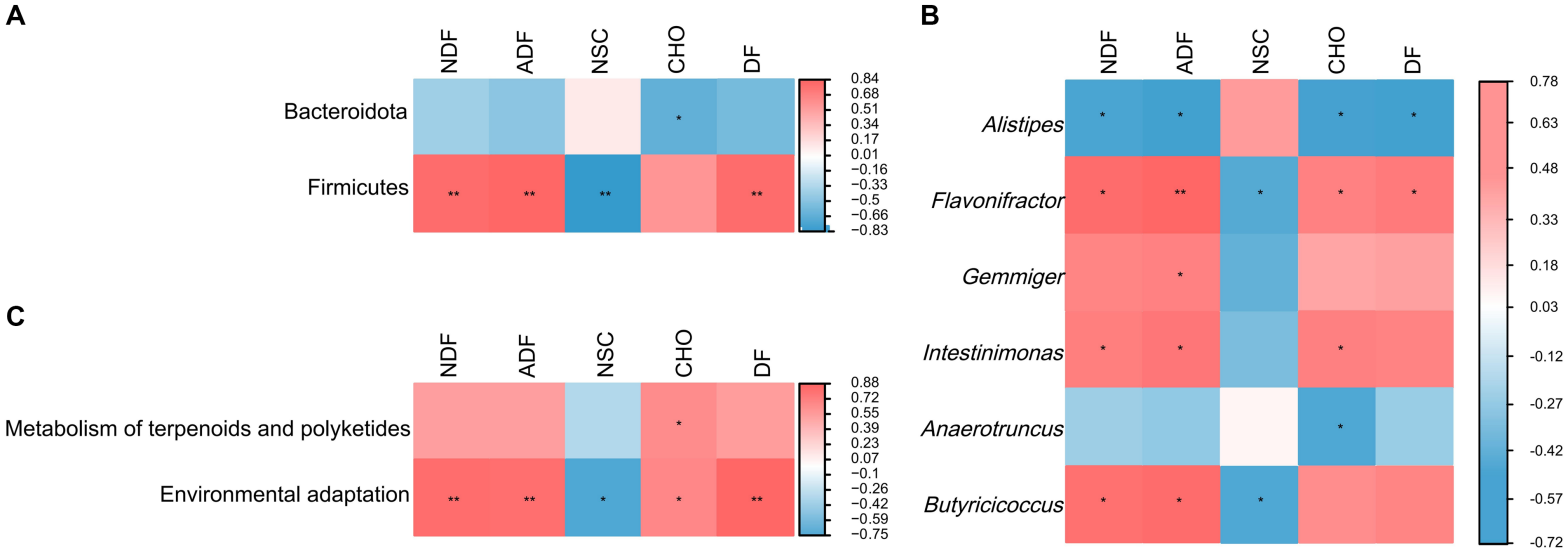
The relationship between feeding and gut microbes. **(A)** microbial phylum associated with plant content intakes; **(B)** microbial genus associated with plant content intakes; **(C)** microbial functions associated with plant content intakes (NDF = neutral detergent fiber; ADF = acid detergent fiber; NSC = non-structural carbohydrate; CHO = carbohydrate; DF = digestible fiber).

The result of random forest analysis showed Firmicutes contributed the most to the differences in gut microbiota and were main biomarker between pre-released and wild red deer (Supplementary Figure S1). Further correlation between feeding and gut microbes showed that the intake of plant fiber was significantly positively correlated with the abundance of Firmicutes (*p* < 0.01, Figure 6A). The intake of plant fiber and carbohydrate significantly affected the abundance of major microbial genera in wild red deer (*p* < 0.05, Figure 6B). Additionally, plant fiber and carbohydrates had significant positive effects on the microbial functions of wild red deer, especially on the expression of environmental adaptive pathway (*p* < 0.05, Figure 6C).

### 4.3 Identification of the main metabolic functional microbes

**Figure 7:**
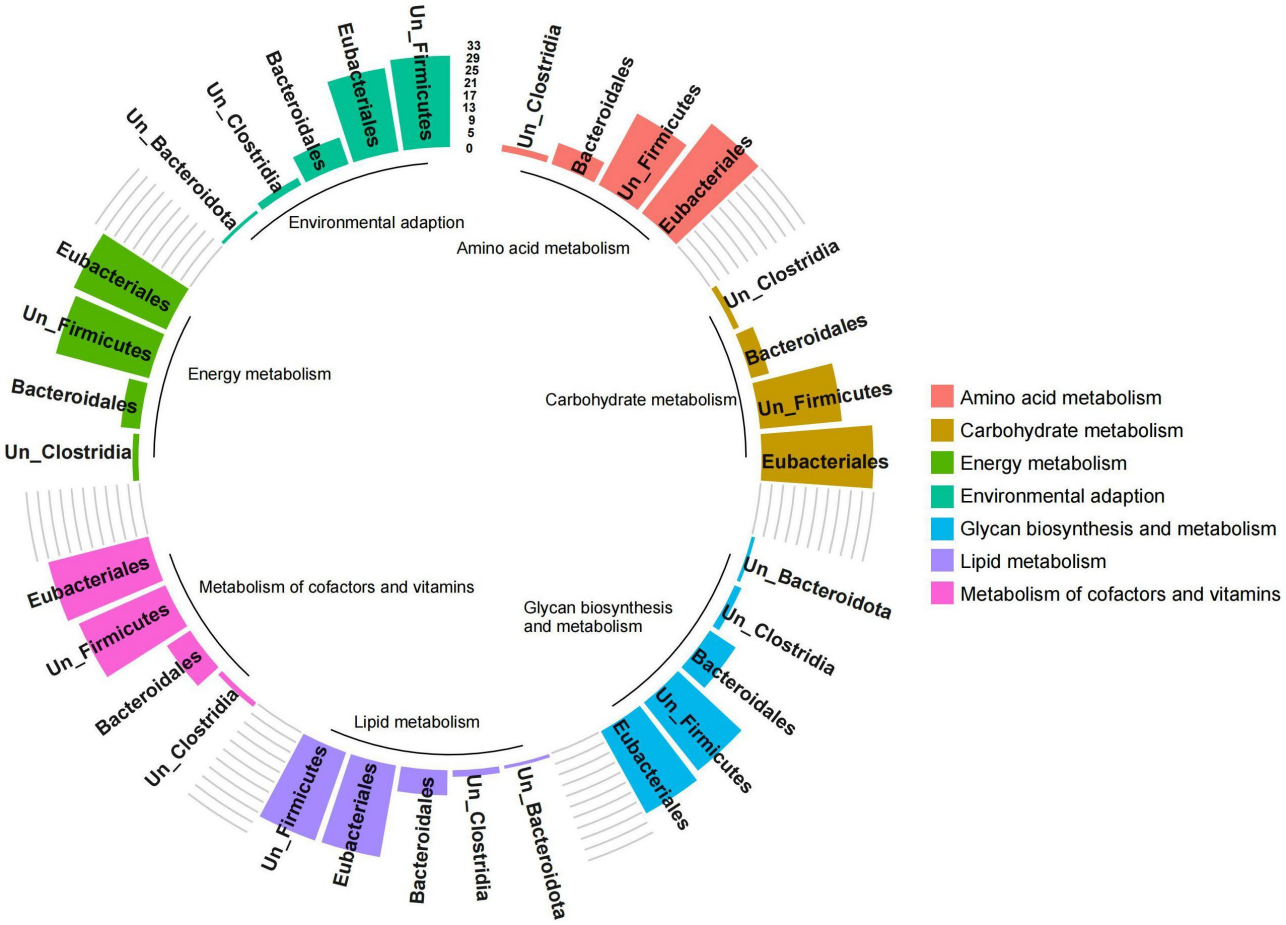
Analysis of functional contribution of gut microbes of red deer. The height of the bar chart represents the functional contribution of different microbial communities. Colors represent different important functional pathways.

The functional contribution of microorganisms was further analyzed based on the functional expression of gut microbes. However, due to some ungraded microbial components at the family and genus levels, only the analysis results at the order level were presented. The results showed that the pathways were mainly related to Eubacteriales, Bacteroidales, unclassified-Firmicutes and unclassified-Clostridia (*importance* > 1%; Figure 7). Among them, the expression of carbohydrate metabolism, amino acid metabolism, glycan biosynthesis and metabolism, metabolism of cofactors and vitamins, energy metabolism, lipid metabolism, and environmental adaptation was significantly positively correlated with the abundance of Eubacteriales, unclassified-Firmicutes, and unclassified-Clostridium (*p* < 0.05, Supplementary Figure S2). These results indicated that Firmicutes represented by Eubacteriales and Clostridia played an important role in regulating microbial metabolism.

It could be seen from the results that the combinations of Firmicutes represented by Eubacteriales and Clostridia, lacking in the gut of pre-released red deer, were important metabolic functional microbes for processing high-fiber plant resources in the wild.

## 5 Discussion

The pre-released red deer are derived from the captive population. Comparing the difference in gut microbes between pre-released and wild red deer, we can understand the adaptability of pre-released red deer to the wild feeding environment (Cheng et al., 2020). In this study, the microbial difference between pre-released and wild red deer was mainly due to Firmicutes, and the abundance of Firmicutes was significantly lower in pre-released red deer. This difference in abundance should be related to feeding conditions. Although the pre-released red deer received some training in feeding, they still primarily fed on corn, soybean meal and green hay. In contrast, the feeding of wild red deer was rich in plant fiber and carbohydrates. Firmicutes with high abundance might promote the degradation of plant fiber by encoding proteases (Flint et al., 2015; Zhao et al., 2018). Clostridia was representative decomposing bacteria of Firmicutes, which could promote the decomposition and utilization of carbohydrates and complex polysaccharides (Aristilde, 2017). The difference in clostridia between pre-released and wild red deer indicted that clostridia might play a key role in the treatment of wild plant resources. However, due to the complexity and difficulty of the wild release, the number of pre-released individuals in this study was limited. To increase the robustness of the results, this study was compared and discussed with the previous research related to wild release of red deer. In 2021, we released 6 red deer individuals into the wild into the Huangnihe National Nature Reserve, and compared with the results of this study, we found that the abundance of Firmicutes in red deer released in 2021 (68.94%) was similar to that of the pre-released individuals (68.23%), and the abundance of Clostridia was significantly lower than that in the wild red deer (Guo et al., 2022). The results of this study were consistent with the results of previous studies, further indicating that the differences in the Firmicutes represented by Clostridia between pre-released and wild red deer. The feeding conditions should be responsible for the difference in gut microbes (Zhen et al., 2022; Sun et al., 2023).

The feeding condition of pre-released red deer was significantly different from that in wild red deer. Due to the nutritional balance and high availability, shrubs became the main food source for wild red deer (Zhong, 2021). The feeding process of wild red deer was accompanied by the intake of high-fiber substances (mainly neutral detergent fiber in the cell wall), but the red deer itself could not directly decompose the high-fiber components in the plant wall to obtain internal nutrients, and needed to rely on the fiber-degrading bacteria in the intestine (Li et al., 2015; Wang et al., 2021). Firmicutes dominated the intestinal tract of cervids and contain a variety of cellulose-degrading bacteria (Kameshwar and Qin, 2016). On the one hand, the feeding of red deer provided a suitable intestinal environment for the colonization of Firmicutes. On the other hand, high levels of Firmicutes could promote the decomposition of plant fibers and the conversion of nutrients. In this study, we further clarified the relationship between feeding and Firmicutes abundance of red deer: the intakes of plant fiber and carbohydrates were positively correlated with Firmicutes abundance. Increasing the intake of plant fiber and carbohydrates could promote maintaining high levels of Firmicutes in the gut (Sun et al., 2022). In studies of the gut microbes of cervids such as roe deer (*Capreolus pygargus*) and moose (*Alces alces*), it has also been pointed out that the high abundance of Firmicutes might be related to adaptation to high-fiber diets (Chen et al., 2022; Yuan et al., 2022). Therefore, the difference of plant fiber and carbohydrate content in feeding was the main reason for the difference in Firmicutes between pre-released and wild red deer. And Firmicutes was important microorganisms for processing plant fiber, carbohydrate, and other macromolecular substances in feeding plants of red deer. In this study, we used the combination of modern microbiology technology and nutritional ecology analysis to innovatively reveal the importance of Firmicutes in red deer to adapt to high-fiber feeding conditions in the wild.

The feeding conditions affect the microbial structure and composition, which in turn has an impact on the microbial functions, especially the metabolic functions (Muegge et al., 2011; Flint et al., 2015), and the changes in gut microbiota can affect the nutrient utilization (Wu et al., 2017; Zhao et al., 2024). In this study, we found that the expressions of carbohydrate metabolism, amino acid metabolism, glycan biosynthesis and metabolism, metabolism of cofactors and vitamins, energy metabolism, and lipid metabolism of wild red deer were significantly higher than those of pre-released red deer. Interestingly, wild red deer had higher expression of amino acid metabolism, but in fact they had a lower crude protein intake and Bacteroides abundance. The asymmetry between the structural diversity and functional niches of gut microbes may be responsible for this phenomenon (Phillips et al., 2017; Xiao et al., 2019). The results of microbial functional contribution indicated that Eubacteriales, Bacteroidales, unclassified Firmicutes, and unclassified Clostridia contributed the most to functional pathways of nutrient metabolism and environmental adaptation, and the functional contribution of the Firmicutes combinations was much greater than that of the Bacteroides, which implied the non-consistency between microbial community and function in red deer. In this study, the expression of metabolism and environmental adaptation pathways had positive relationships with the abundance of Eubacteriales, unclassified Firmicutes, and unclassified Clostridia, indicating that high levels of Firmicutes combinations could promote the expression of microbial functional pathways. The relationship between Firmicutes and microbial metabolic function has also been illustrated in other studies, an increase in carbohydrate intake of moose significantly increased the abundance of Firmicutes, demonstrating the important function of Firmicutes in carbohydrate metabolism (Bao, 2019). In addition, Firmicutes are associated with fat deposition and energy absorption in animals, and increasing the abundance of Firmicutes can regulate lipid metabolism and promote the energy conversion, which plays an important role in meeting the behavioral needs of animals and maintaining normal body temperature (Bäckhed et al., 2004; Ley et al., 2006). Therefore, Firmicutes, represented by Eubacteriales and Clostridia, were critical bacteria of red deer to cope with feeding plant resources and regulate metabolic functions in the wild. However, sequencing technology limited the interpretation of the results and could not deeply classify Firmicutes. In the future, more sophisticated technologies should be used.

In summary, the relationship between feeding and gut microbes of red deer was shown in that the increase of plant fiber and carbohydrate intake would significantly affect the abundance of Firmicutes, and then affect the expression of metabolic functional pathways. The combinations of Firmicutes were important microbial groups for processing plant resources in the wild, and the low level of Firmicutes in the gut of pre-released red deer might affect the adaptability to natural feeding environment after release. Therefore, in the wild release of red deer, more attentions should be paid to the number of Firmicutes represented by Eubacteriales and clostridia in the gut. The adaptability of pre-released deer to wild plant resources could be improved by increasing the feeding of wild plants. Future studies should pay more attention to the adaptability to the wild environment of wild released red deer, especially the relationship between the number of Firmicutes and the feeding adaptability.

## 6 Conclusion

Firmicutes are the main biomarkers and differential microflora between pre-released and wild red deer. The combinations of Firmicutes play important roles in regulating microbial metabolic functions and maintaining gut homeostasis, and are key microflora that helps red deer adapt to the wild environment. The differences in gut microbes between pre-released and wild red deer may affect the habitat adaptability of red deer after wild release. It is suggested to increase the proportion of wild plants in the food components of pre-released red deer to promote the colonization of Firmicutes in gut. After release into the wild environment, the gut microbes and wild adaptation should be further monitored and investigated.

## Data availability statement

The raw sequences of metagenomic sequencing for this study can be found in the Sequence Read Archive from NCBI. Bio project number: PRJNA1087561. Link: https://dataview.ncbi.nlm.nih.gov/object/PRJNA1087561?reviewer=qvab6e3o4tp7c801gsdgnhc6qs.

## Ethics statement

Ethical approval was not required for the study involving animals in accordance with the local legislation and institutional requirements because the subjects of our study were red deer living in forested areas, and we used non-invasive fecal analysis techniques, all of which were approved and supported by local authorities.

## Author contributions

JG: Writing – original draft, Visualization, Validation, Software, Resources, Methodology, Investigation, Formal Analysis, Data curation. ZL: Writing – review & editing, Software, Resources, Investigation, Formal Analysis. YJ: Writing – review & editing, Visualization, Software, Formal Analysis. YS: Writing – review & editing, Validation, Software, Methodology. BW: Writing – review & editing, Investigation. XL: Writing – review & editing. ZY: Writing – review & editing. WZ: Writing – review & editing. CZ: Writing – review & editing, Visualization, Supervision, Funding acquisition. MZ: Writing – review & editing, Validation, Supervision, Project administration, Funding acquisition, Conceptualization.

## Supplementary material

Supplementary Table S1 The information of pre-released red deer.

Supplementary Figure S1 The random forest analysis between pre-released and wild red deer.

Supplementary Figure S2 The relationship between the expression of microbial functions and microbial abundance. Different colors represent microbial communities.
