# Supplementary material for: The gut microbial differences between pre-released and wild red deer: Firmicutes abundance may affect wild adaptation after release
Source: Front Microbiol. 2024 Jul 15;15:1401373. doi: 10.3389/fmicb.2024.1401373 (PMC11284171; doi:10.3389/fmicb.2024.1401373)
Supplement: Supplementary Table S1 — The information of pre-released red deer. [file Table_1.DOCX]

Table. S1 The information of pre-released red deer

| **Numbering** | **Gender** | **Age** | **Food composition** | **Health status** |
| --- | --- | --- | --- | --- |
| Pre-released 1 | male | 5.5 | corn, soybean meal, green hay, wild shrubs and herbs | good |
| Pre-released 2 | female | 5.0 | corn, soybean meal, green hay, wild shrubs and herbs | good |
| Pre-released 3 | female | 5.0 | corn, soybean meal, green hay, wild shrubs and herbs | good |
