# Supplementary figures and images for: The gut microbial differences between pre-released and wild red deer: Firmicutes abundance may affect wild adaptation after release
Source: Front Microbiol. 2024 Jul 15;15:1401373. doi: 10.3389/fmicb.2024.1401373 (PMC11284171; doi:10.3389/fmicb.2024.1401373)

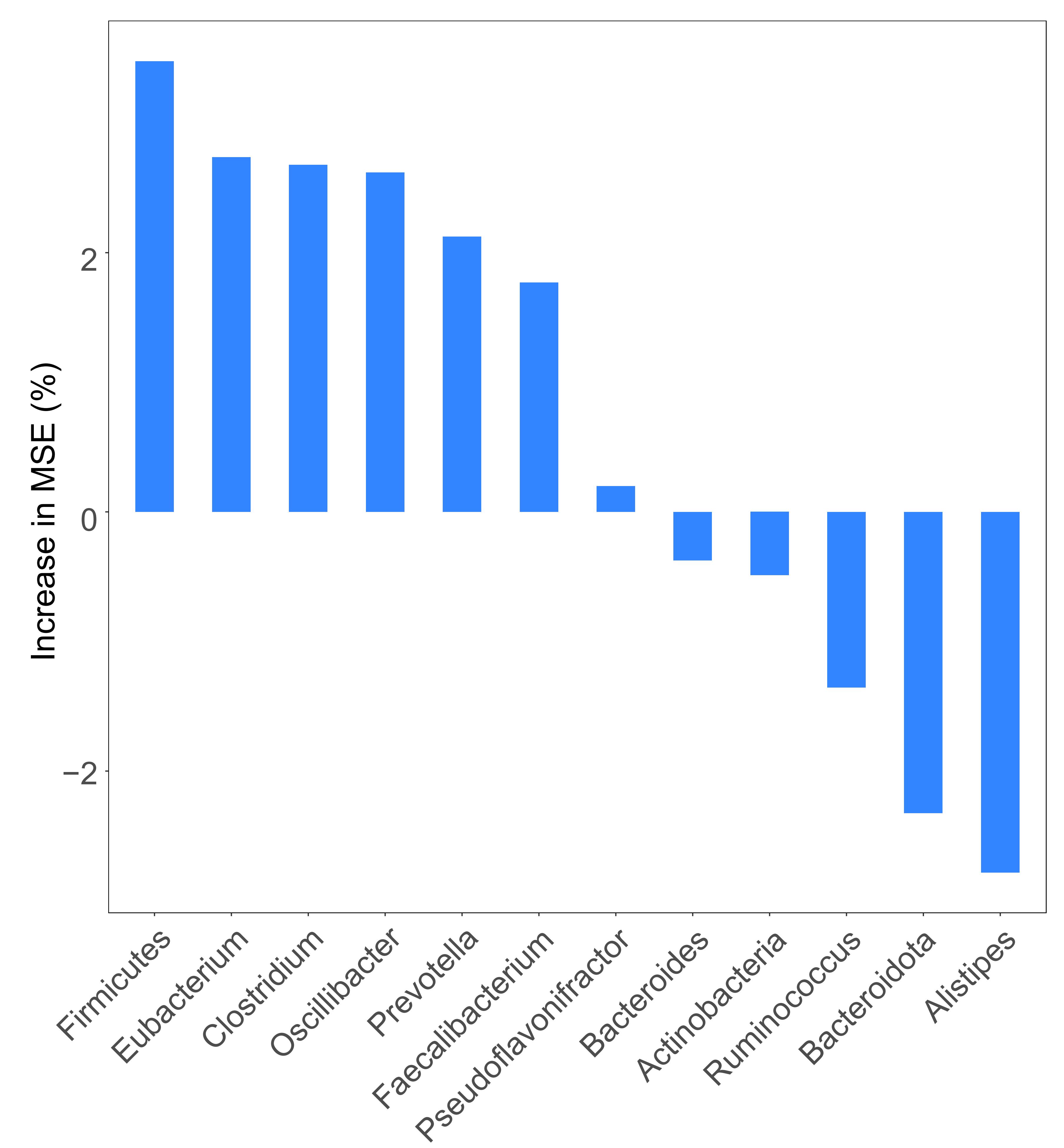

Supplement: Supplementary Figure S1 — The random forest analysis between pre-released and wild red deer. [file Image_1.JPEG]

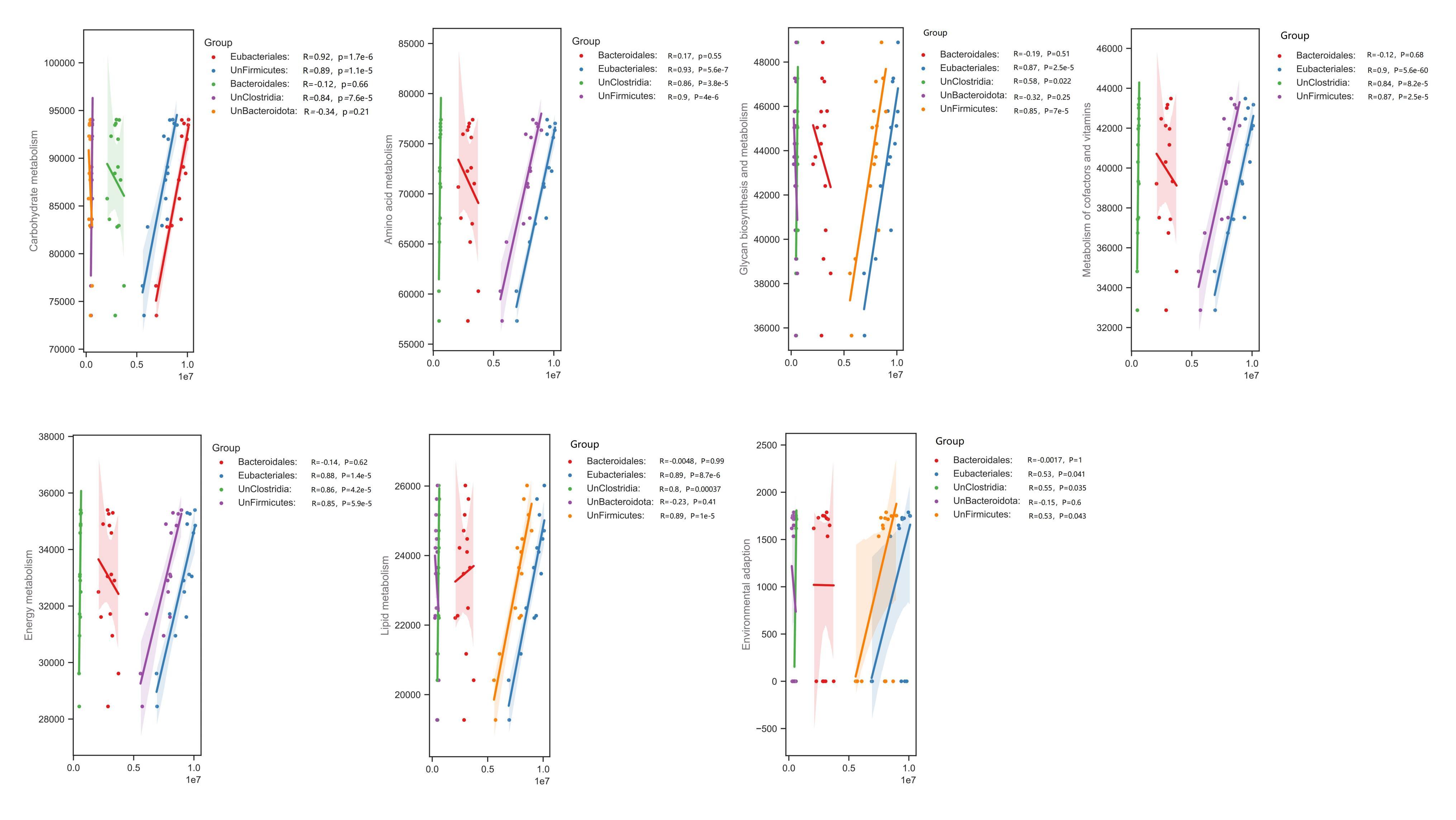

Supplement: Supplementary Figure S2 — The relationship between the expression of microbial functions and microbial abundance. Different colors represent microbial communities. [file Image_2.JPEG]
